# Inhibitory Circuits Can Restore OFF Pathway Responses in Retinal Prostheses

**DOI:** 10.1523/JNEUROSCI.0083-26.2026

**Published:** 2026-05-14

**Authors:** Maya Carleton, Nicholas W. Oesch

**Affiliations:** ^1^Departments of Psychology, University of California San Diego, La Jolla, California 92093; ^2^Ophthalmology, University of California San Diego, La Jolla, California 92093; ^3^Neuroscience Graduate Program, University of California San Diego, La Jolla, California 92093

**Keywords:** brain–machine interface, neural circuits, neural prosthetics, retina, retinal disease, vision restoration

## Abstract

One of the first steps in processing visual information is to split the light signal captured by photoreceptors into complementary ON and OFF pathways, which separately encode increases and decreases in luminance. In blind patients with retinal degeneration, optoelectronic prostheses can successfully activate the ON pathway and evoke bright percepts; however, patients do not perceive dark features. This indicates that the OFF pathway is not being activated by existing prosthetics. To quantify OFF pathway deficits, we stimulated retinal ganglion cells (RGCs) in a mouse model of retinitis pigmentosa of either sex with electrical stimulation mimicking retinal prosthetic activation and recorded their voltage responses using whole-cell recording. We found that most OFF RGCs respond with incorrect ON responses, except for one specific subtype of RGC, the OFFα, which retained correct OFF-type responses following the termination of stimulation. We found that these preserved OFF responses were driven by postinhibitory rebound excitation, mediated by hyperpolarization-activated cyclic nucleotide-gated channels. Using a combinatorial genetic approach to achieve chemogenetic control, we identified AII amacrine cells as the presynaptic source driving these electrically evoked OFF responses. These insights into how the OFF pathway responds to artificial stimulation suggest new opportunities to improve prosthetic vision restoration through tuning of stimulation parameters.

## Significance Statement

Retinal prostheses in blind patients have thus far been able to recreate the perception of bright objects, corresponding to the activation of the ON pathway in the retina. However, patients can not perceive dark features, signals that should be carried by the retinal OFF pathway. Complementary ON and OFF signaling is crucial for normal visual perception. This study identifies key retinal circuitry that, when stimulated with electrical pulses, can elicit proper OFF pathway responses in a key subtype of retinal ganglion cell. We identify the physiological and circuit mechanisms that underlie these restored OFF responses. These findings will guide development of targeted stimulation of the OFF pathway in future therapies to improve restored vision in patients with retinal degenerated diseases.

## Introduction

Age-related macular degeneration (AMD) and retinitis pigmentosa (RP) are the leading causes of noninherited and inherited blindness, respectively, resulting in ∼200 million cases of untreatable blindness worldwide (Wong et al., 2014; Bourne et al., 2017). Blindness in AMD and RP results from degeneration of light-sensing photoreceptors, while inner retinal neurons remain intact. The goal of vision restoration is to restore purposeful vision to patients with retinal degenerative diseases. Retinal prosthetic devices and optogenetic gene therapies—forefront restoration techniques—aim to restore visual function by translating light into stimulation of the remaining retinal circuitry. Retinal prosthetics achieve this via optoelectronic hardware (Zrenner et al., 2011; Daschner et al., 2018; Holz et al., 2025), while optogenetic therapies utilize the transfection of genetically encoded light sensitive proteins (Dowling, 2009; Simunovic et al., 2019; Lindner et al., 2022). Notably, both therapies rely on the survival of inner retinal neurons to act as a substrate for the restoration of visual inputs.

In the healthy retina, photons are converted into electrochemical signals by photoreceptors. Increments or decrements in photon flux are separately encoded by two parallel pathways, the ON and OFF pathways, which originate in ON and OFF bipolar cells, that differentially respond to glutamatergic output from photoreceptors (Schiller, 1992; Masland, 2001; Wässle, 2004). These pathways function synergistically to rapidly and efficiently produce vision across a wide range of lighting conditions (Schiller, 1992; Mazade et al., 2019; Ichinose and Habib, 2022). While it is clear that electrical and optogenetic stimulation can generate artificial ON-type responses at both the cellular and perceptual level (Ho et al., 2018; Palanker et al., 2020; Sahel et al., 2021; Khabou et al., 2023; Holz et al., 2025), how artificial stimulation interacts with the OFF pathway remains incompletely understood. The general dogma assumes that electrical stimulation should equally stimulate both ON and OFF-type bipolar cells, which will generate simultaneous ON-type responses in both the ON and OFF pathways (Goetz et al., 2015; Werginz et al., 2015; Goetz and Palanker, 2016; Golden et al., 2019; Paknahad et al., 2022), although the extent to which this is true in degenerated retina has not been rigorously tested. This lack of understanding about this fundamental visual signaling pathway significantly impedes our ability to design better retinal prosthetics for retinal degenerative diseases affecting photoreceptors.

To address these outstanding questions about how artificial visual stimulation interacts with the OFF pathway, we used single-cell current-clamp recordings to measure electrically evoked action potentials of morphologically identified ON and OFF retinal ganglion cells (RGCs). For the first time, we quantified the frequency of ON versus OFF responses in identified OFF RGCs in degenerated retina and found that most OFF RGCs do not respond to a single strong electrical pulse, unlike ON RGCs, but they can be driven via high-frequency pulse trains. Importantly, we found that when they do respond, the majority of OFF RGCs respond with an ON functional response. There was a minority of OFF RGCs that did respond with a “correct” OFF response at the termination of electrical stimulation, which we identified were the OFFα RGC subtypes. We found these electrical OFF responses were due to postinhibitory rebound excitation (PIRE) mediated by inhibition from the AII network. These results show the complex interactions of electrical stimulation within the ON and OFF pathways in the degenerated retina. This work directly points to stimulation protocols that can selectively activate a subset of the OFF pathway in future retinal prosthetic devices.

## Materials and Methods

### Retina explant and voltage-clamp recording

All experimental methods and animal care procedures were conducted in accordance with National Institutes of Health guidelines and were approved by the University of California, San Diego Institutional Animal Care and Use Committee. Forty-one adult (PND 65-207, mean = 109 ± 34 PND, median = 115) RD10 mice of either sex were anesthetized with isoflurane, killed by cervical dislocation, and enucleated, and their retinas were dissected free and maintained in Ames medium oxygenated and equilibrated with 95% O_2_, 5% CO_2_. Retina pieces, ∼2 mm × 2 mm, were transferred to a custom recording chamber and placed over stimulating electrodes on the bottom of the custom recording chamber, ganglion cell side up. We used retinal pieces from all regions of the retina, without targeting any quadrant or eccentricity. The chamber was placed under an upright microscope and perfused with Ames (Ames and Nesbett, 1981) media (United States Biological) at a rate of 4 ml/min at 35°C. RGCs were visualized and targeted using IR differential interference contrast (IR-DIC) video microscopy. Contact between the outer portion of the retina and the electrode array surface was also confirmed with microscopy. Stimulation was delivered on the nearest electrode to the target RGCs. For AII amacrine cell recordings, mice carrying the CDH1-eGFP allele Tg(Cdh1-EGFP)AR201Gsat/Mmucd; RRID:MMRRC_011775-UCD (Gong et al., 2003) were bred onto the rd10 line so that AII amacrine cells could be identified based on GFP fluorescence (Firl et al., 2015).

Sputtered iridium oxide film (SIROF) stimulating electrode arrays were fabricated as described previously on a borosilicate glass disk (Nano3, San Diego Nanotechnology Infrastructure of UCSD), which formed the bottom of the recording chamber (Damle et al., 2021). Briefly, the array consisted of a 4 × 8 grid of 30 µm diameter SIROF electrodes spaced 50 µm apart from center to center. SIROF electrodes were formed by reactive DC sputtering to a thickness of 600 nm over indium tin oxide (ITO) traces. ITO traces terminated in gold contact pads at the edge of the disk for connection to a 32-channel RHS2000 stim and recording system (Intan Technologies). Charge-balanced, anodic first, square, biphasic current pulses were generated on the RHS2000, triggered by our acquisition software, and delivered to an individual stimulating electrode nearest to the cell of interest. Arrays of 30 µm diameter iridium oxide electrodes were used to stimulate the inner nuclear layer with 1 ms, 20 µA biphasic anodic first pulses (20 nC per phase). Distances were measured using Scientifica LinLab2 and IR-DIC microscopy. The cell was positioned in the center of the field of view and marked as the coordinate zero point. The stage was then moved to the center of the stimulating electrode, and the *x*–*y*-coordinates from LinLab2 was recorded. Stimulation was always delivered on the nearest stimulating electrode, given the 50 µm square spacing of the electrode array, this means the target cell center was always 35 µm or less from the center of the stimulating electrode.

Recording electrodes were pulled from borosilicate capillary glass to have a final resistance of 4–5 MΩ for RGC recordings and 6–7 MΩ for AII amacrine cell recordings. For current-clamp recordings, electrodes were filled with internal solution composed of the following (in mM): 90 K-MeSO_4_H, 20 KCl, 10 EGTA, 10 HEPES, 10 phosphocreatine disodium salt hydrate, 4 Mg-ATP, and 0.4 Na-GTP. For voltage-clamp recordings, electrodes were filled with internal solution composed of the following (in mM): 90 CsCH_3_SO_3_, 20 TEA-Cl, 10 HEPES, 10 EGTA, 10 phosphocreatine disodium salt, 4 Mg-ATP, and 0.4 Na-GTP. Whole-cell voltage- or current-clamp recordings were made from ganglion or amacrine cells using a MultiClamp 700B (Molecular Devices) patch-clamp amplifier in current-clamp mode. Signals were filtered at 4 kHz (4-pole Bessel), digitized at 20 kHz with an ITC-18 (HEKA Elektronik) data acquisition board, and saved to a PC for offline analysis using custom acquisition software in Igor Pro 8 (WaveMetrics).

For experiments where pharmacological agents were used, these agents were added to the superfusate. Excitation was blocked with 10 µM NBQX and 50 µM AP5 (Tocris BioScience) to block AMPA/kainite and NMDA receptors, respectively. Glycinergic neurotransmission was blocked with 5 µM strychnine (Sigma-Aldrich). Hyperpolarization cyclic nucleotide (HCN) gated ion channels were blocked using 20 µM ZD7288 (Sigma-Aldrich). For experiments involving the DREADD hM4D(Gi), 10 µM clozapine *N*-oxide (CNO) was used as an agonist for hM4Di receptors (Bio-Techne/R&D Systems).

### Cell identification

RGCs were labeled for morphological analysis using an electrode containing internal solution with the addition of 2% Lucifer yellow or Alexa 488 (LY, Invitrogen). Whole-cell current-clamp recordings were made to observe the functional response properties. During the recording, cells were filled by passive diffusion. The retina was then removed from the recording chamber and fixed in 4% paraformaldehyde in 0.1 M phosphate buffer (pH 7.4) for 45 min. Retinas were then washed in 0.1 M phosphate buffer for 25 min before being placed in 3% agarose (Sigma-Aldrich). Agarose blocks containing retina were oriented vertically on a PELCO easiSlicer (Ted Pella) and sliced at 200 µm. Sliced retina was plated and imaged on an epifluorescence microscope (Olympus BX36). The inner and outer boundaries of the inner plexiform layer (IPL) were determined by the ganglion cell layer and the inner nuclear layer visible with bright-field illumination. Cells were classified as ON, OFF, or ON/OFF based on dendrite depth within the IPL. Dendritic arbors were traced using NeuronJ (ImageJ, National Institutes of Health).

To confirm identify of OFFα RGCs, following fixation and washes as described above, retinas were incubated in anti-SMI-32 antibody conjugated to an Alexa 594 fluorophore (BioLegend) for 2 h at room temperature (5 μg/ml). Following incubation, retinas were washed an additional three times and mounted in whole-mount configuration for imaging. Colocalization of Alexa 488 and Alexa 594 was confirmed using ImageJ (National Institutes of Health).

### Virus and intravitreal injections

AAVs were obtained from Addgene (pAAV-EF1a-Flp-DOG-NW was a gift from Connie Cepko; Addgene viral prep #75469-AAV1; http://n2t.net/addgene:75469; RRID: Addgene_75469; Tang et al., 2016), pAAV-hSyn-fDIO-hM4D(Gi)-mCherry-WPREpA was a gift from Ulrik Gether (Addgene viral prep #154867-AAV8; http://n2t.net/addgene:154867; RRID:Addgene_154867; Runegaard et al., 2018). Virus was injected intravitreally into adult RD10-CDH1-eGFP mice anesthetized with isoflurane and secured to a stereotaxis. A 27 G hypodermic needle was used to puncture the eye at the limbus before a 30 G blunt Hamilton syringe was advanced into the intravitreal space. Then, 2.5 µl of each virus was injected intravitreally in each eye. Transfection time was between 10 and 12 d post injection, at which point injected mice were killed and retinae were excised as detailed above.

### Analysis

Data processing and statistical analyses were performed in Igor Pro 8. Spikes were identified by thresholding the first derivative of the raw data with a threshold that was >5× the peak-to-peak noise amplitude. Individual cell responses were calculated from the average of the five repeats. To remove stimulus artifacts from spike counts, detected event times with a peak time within 200 µs of the stimulus artifact peak were discarded. For cells with high basal activity, the spontaneous action potential count for a time window prior to the stimulus was subtracted from the spike count to isolate stimulus-evoked activity. Peristimulus time histograms (PSTHs) were calculated using the instantaneous spike rate from the interspike interval. Reported values are the mean ± 1 SD. We used a Student’s *t* test (paired and unpaired) for comparison of two groups. For comparison of multiple groups, we used a one-way ANOVA with Tukey HSD post hoc test to correct for multiple comparisons. Significance levels were set as follows: *p < 0.05, **p < 0.005, and ***p < 0.0005. Box plots use the Tukey 1st quartile, median, and 3rd quartile; whiskers represent 1 SD, and yellow diamonds represent the mean.

Conductance analysis was performed in a similar method to previous studies (Borg-Graham, 2001; Oesch and Taylor, 2010). Stimulus-evoked responses were recorded at 13 holding potentials (from −100 to +20 in 10 mV steps. *I*–*V* relationships were measured at 5 ms intervals for the duration of the voltage step, relative to the baseline in the preceding 500 ms. Voltage series were repeated two times and averaged. Synaptic currents were assumed to arise from a sum of linear excitatory and inhibitory synaptic inputs. Excitation is mediated by nonselective cation channels with a reversal potential of 0 mV, while inhibition is mediated by chloride channels with a reversal potential of −54 mV. Each *I*–*V* was fit with a line between −80 and−40 mV where the *I*–*V* relation was most linear. The slope and intercept were determined from the fit, producing a discrete measurement of conductance at every point.

## Results

**Figure 1.**
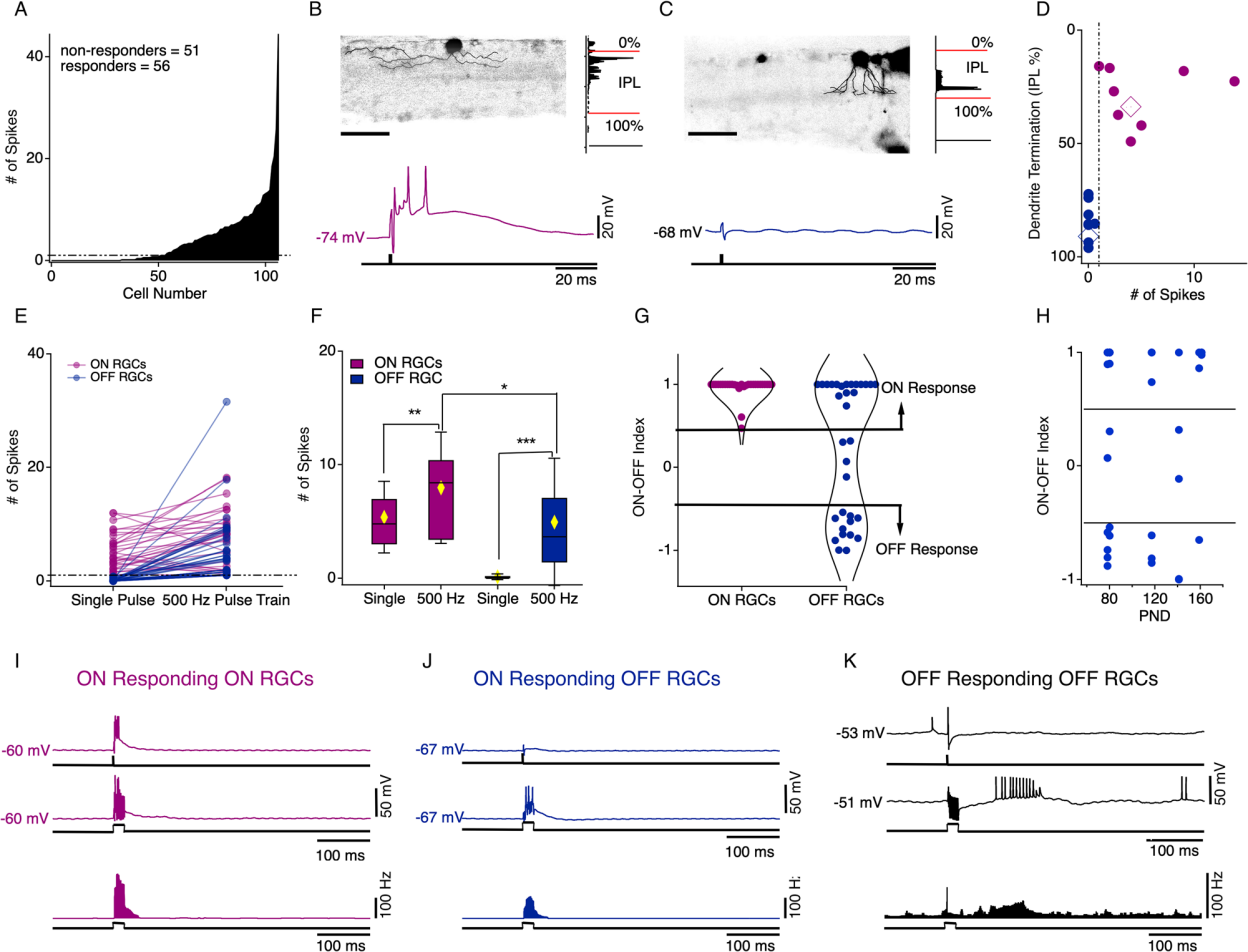
RGC responses to single biphasic pulses. ***A***, Average stimulus-evoked spikes per trial for 107 cells. ***B***, Example dendritic morphology with voltage recording for a responding and nonresponding cell. ***C***, Right inset, Histogram of pixels in the IPL from 0 to 100%. Scale bar, 50 µm. ***D***, Dendritic termination depth percentage versus average number of spikes for filled cells for responders (magenta) and nonresponders (blue). Diamonds correspond to examples in ***B***, ***C***. ***E***, Within cell comparison of spiking in response to a single pulse and 500 Hz pulse train for ON RGCs (magenta, *n* = 29) and OFF RGCs (blue, *n* = 43). Horizontal dashed line is at responding threshold (1 spike). ***F***, Comparison of stimulus-evoked spikes for single pulse and 500 Hz pulse train in ON cells and OFF cells. ***G***, Violin plot of ON–OFF response index for ON cells and OFF cells. Black lines at 0.5/−0.5 denote ON and OFF response threshold, respectively. ***H***, ON–OFF index for OFF RGCs plotted against age. ***I***, Example current-clamp trace for a classified ON-responding ON RGC to a single pulse (top) and 500 Hz pulse train (middle). Average spike rate for all ON-responding ON cells (bottom). ***J***, Same as ***I*** but for ON-responding OFF RGCs. ***K***, Same as ***I*** but for OFF-responding OFF RGCs. Significance levels were as follows: *p < 0.05, **p < 0.005, and ***p < 0.0005, Student’s *t* test (***F***). Box plots use the Tukey 1st quartile, median, and 3rd quartile, whiskers represent 1 SD, and yellow diamonds represent the mean. For entire the figure, *n* = 27 animals (PND 70–161), mean age 112 ± 30.

Prior work assessing the fidelity of optoelectronic signals in the photoreceptor degenerated retina has primarily been done using MEAs or loose patch recordings (Boinagrov et al., 2014; Stutzki et al., 2016; Ho et al., 2018; Carleton and Oesch, 2022), which cannot resolve cells that do not reach spiking threshold in response to the electrical stimulus. In general, it is widely assumed that electrical stimulation of the remaining inner retina will simultaneously stimulate both ON and OFF RGCs, although this has not been systematically tested in photoreceptor degenerated retina. Using the whole-cell current-clamp technique, we recorded electrical stimulation-evoked action potentials following a stimulation pulse. This allowed us to record both responsive and nonresponsive RGCs. We chose to initially use a single biphasic stimulation pulse at 20 nC which is at the safe injection limit for a 30 µm SIROF electrode based on the charge injection capacity (Damle et al., 2021). While many cells fired action potentials in response to a 20 nC stimulation pulse as expected (Damle et al., 2021), we observed many cells that did not respond. We also observed a proportion of RGCs that showed spontaneous firing, as has previously been described (Trenholm and Awatramani, 2015). However, this was a minority of the cells we collected with ∼26% having high basal firing rates. To quantify evoked spikes in spontaneously active cells, we measured baseline firing in the 500 ms proceeding the electrical stimulus and subtracted this from evoked spiking in the 500 ms following the electrical stimulus. We defined an electrically responding RGC as one that had one or more spikes above baseline for the average of five trials. We found that approximately half of the sampled RGCs did not fire an action potential to a single stimulation pulse (responders, *n* = 56; nonresponders, *n* = 51; Fig. 1*A*). This indicates that many RGCs do not respond to single electrical stimulation pulses and suggests past work with extracellular recordings may have been biased toward responding RGCs and not representative of entire RGC population.

In our prior work assessing the E/I balance in ON and OFF RGCs in the rd10 retina, we found decreased excitatory drive in OFF RGCs relative to wild-type, but not ON RGCs (Carleton and Oesch, 2024). We hypothesized that this could limit action potential generation in OFF RGCs following an electrical stimulus. To date, no study has measured electrical spiking responses in morphologically identified RGCs in photoreceptor degenerated retina. To find the morphological ON/OFF identity of responding and nonresponding cells, we filled a subset of cells with Alexa 488 during whole-cell recording. In this subset, all responding cells had dendrites in the inner half of the IPL, consistent with ON cell morphology and an average spike response of 7.5 ± 7.1 spikes (*n* = 9; Fig. 1*B*,*D*). In contrast, all nonresponding RGCs had dendrites in the outer half of the IPL consistent with OFF cells morphology and an average spike response of 0 ± 0.06 (*n* = 10; Fig. 1*C*,*D*). Therefore, we subsequently used a single 20 nC pulse to physiologically classify ON versus OFF RGCs. These results show a clear response bias present in ON versus OFF RGCs.

### Response to high-frequency pulse train stimulation

The extent to which OFF RGCs respond to electrical stimulation and the timing of those responses has important implications for how retinal prosthetics could reintroduce normal signaling pathways. While single pulses effectively discriminated between ON and OFF RGCs, it is possible that OFFs may be driven by stronger stimuli, such as pulse trains. To determine how pulse trains stimulate ON and OFF RGCs, we delivered 10, 20 nC, biphasic pulses at a frequency of 500 Hz. High-frequency pulse trains elicited spikes in all recorded RGCs, including both responding (ON) RGCs and nonresponding (OFF) RGCs (Fig. 1*E*). Evoked spiking was significantly increased for both ON and OFF RGCs in response to the 500 Hz pulse train compared with a single pulse (paired *t* test: *t*_(28)_ = −3.28, *p* < 0.005 and paired *t* test: *t*_(42)_ = −5.66, *p* < 0.0005, respectively; Fig. 1*F*). For 500 Hz stimulation, ON cells also fired significantly more spikes than OFF RGCs (Fig. 1*F*; *t* test: *t*_(35)_ = −2.41, *p* < 0.05). More robust ON RGC responses compared with OFF RGC responses likely reflect greater E/I ratios in ON versus OFF RGCs (Carleton and Oesch, 2024).

We observed that a majority of both ON and OFF RGCs responded during and immediately following the stimulation pulse train (within 70 ms), consistent with the kinetics of ON responses (Tengölics et al., 2019). This was true for all ON RGCs (29 out of 29) and the majority of OFF RGCs (27 of 43). In addition, we observed a subset of RGCs that did not fire action potential in this ON response window but had a delayed burst of firing consistent with OFF responses to a brief positive contrast stimulus (12 out of 43; Tengölics et al., 2019). To quantify ON versus OFF responses, we calculated a response polarity index (Carcieri et al., 2003; Farrow and Masland, 2011) where pure ON responses have a value of 1 and pure OFF responses have a value of −1 (Fig. 1*G*). For ON classified cells we found a distribution with a mode at 1 and a mean of 0.96 ± 0.12, indicating that ON RGCs have an ON-polarity response to electrical stimulation pulse trains. For OFF classified cells, we found a bimodal distribution with a main mode at 1 and smaller mode at −1, indicative of two response types to high-frequency stimulation for OFF RGCs. When we removed OFF RGCs with response indexes below −0.5, the mean index was 0.85 ± 0.28, which was not significantly different from the ON RGC index (Fig. 1*G*; *t* test: *t*_(23.8)_ = −1.67, *p* = 0.11). To ensure that the presence of ON-responding OFF RGCs was not a function of degeneration state, we plotted the polarity index as a function of age. We did not find any correlation between polarity index and age indicating that ON- and OFF-responding OFF RGCs represent two different populations rather than a change in retinal health or circuit degeneration (Fig. 1*H*). Based on these differences in response properties to single pulses and 500 Hz stimulation, we designated three groups of RGCs for subsequent analysis: ON-responding ON RGCs (magenta), ON-responding OFF RGCs (blue), and OFF-responding OFF RGCs (black; Fig. 1*I–K*). Taken together these results show that the majority of OFF classified RGCs will respond with an ON-polarity to electrical stimulation; however, a smaller subset can maintain OFF-polarity responses [OFF-responding OFF RGCs (black); Fig. 1*K*]. Contrary to the general assumption, these results identify a subpopulation of OFF RGCs that retain an appropriate OFF response to the termination of the electrical pulse train. In addition, we show that the remainder of OFF RGCs does respond to electrical stimulation, although with incorrect ON-type responses. Identifying correctly responding OFF RGCs indicates that some OFF pathway restoration may be possible.

### OFF alpha RGCs maintain OFF functional responses

**Figure 2.**
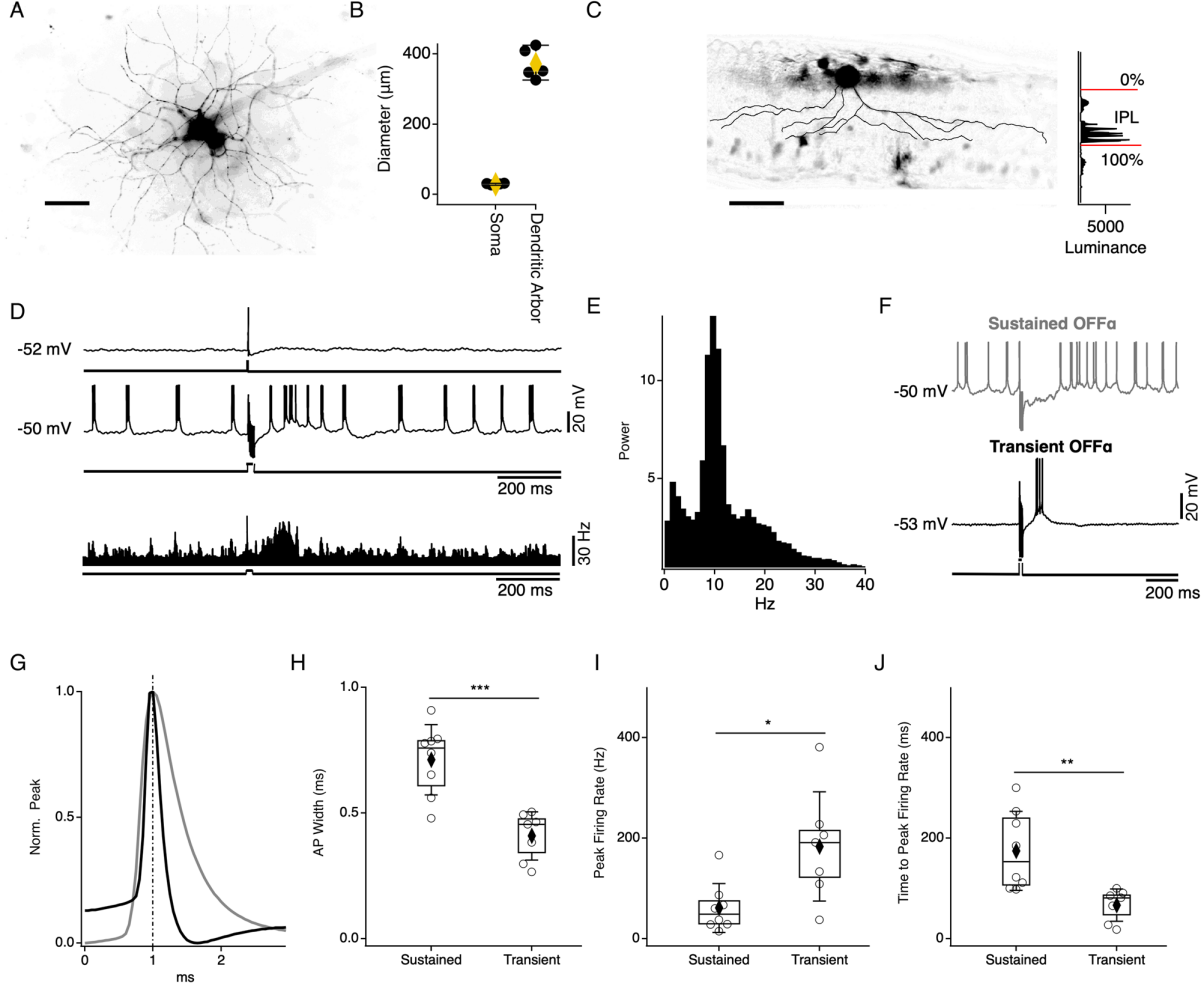
OFF alpha RGCs maintain functional OFF responses. ***A***, RGC filled with Alexa 488 in whole-mount configuration. Scale bar, 50 µm. ***B***, Average soma size and dendritic arbor for filled cells, *n*  =  5, average soma size = 28.75 µm, average dendritic arbor = 371.99 µm. Black dots represent each cell. Yellow diamond represents the average. ***C***, RGC filled LY in vertical slice to show dendritic termination depth in IPL. ***D***, Example current-clamp traces in response to single pulse (top) and 500 Hz stimulation (middle). Average spike rate for all OFFα RGCs (bottom). ***E***, Average PSD for collected cells, *n* = 15. ***F***, Example traces from putative sustained (gray) and transient (black) OFFα RGCs. ***G***, Example peak normalized action potential waveform from a transient and sustained cell. ***H***, Box plot comparison of action potential width in sustained (*n* = 8) and transient (*n* = 7) cells. ***I***, Same as ***H*** but for peak firing rate. ***J***, Same has ***H*** but from time to peak firing rate. Significance levels were as follows: *p < 0.05, **p < 0.005, and ***p < 0.0005. Box plots use the Tukey 1st quartile, median, and 3rd quartile, whiskers represent 1 SD, and black diamonds represent the mean. For the entire figure, *n* = 10 animals (P73–165), mean age = 108 ± 30.

Given the significance of finding electrically evoked OFF responses in OFF RGCs, we were interested in examining this subset of OFF RGCs more closely. The bimodal distribution of the OFF response index suggests that OFF-responding OFF RGCs may represent a subtype of OFF RGCs; however, it is also possible that these OFF responses occur randomly, but infrequently, in the whole OFF RGC population. This could impact the quality of restored vision, because occasional distributed OFF responses would be lost in the averaged OFF pathway signal to the brain, but if a specific OFF pathway retains its OFF signaling, this could appropriately signal OFF information for that specific information pathway. To answer this question, we filled RGCs with Alexa 488 during recording and recovered the morphology of OFF-responding RGCs. We found that OFF-responding OFF RGCs had a large soma (∼30 µm) and dendritic arbor (∼300 µm; *n* = 5; Fig. 2*A*,*B*), consistent with OFFα RGCs (Krieger et al., 2017). Additionally, filled somas colocalized with staining for SMI-32, a marker for α RGCs (Krieger et al., 2017; Fig. S1*A–D*). Physiologically, these cells did not respond to a single electrical pulse, consistent with other OFF RGCs but did have a strong OFF-type burst following a 500 Hz pulse train (Fig. 2*D*; *n* = 15). Furthermore, we found that OFF-responding RGCs had a high basal firing rate with a peak oscillatory power of 8–12 Hz (Fig. 2*E*), also consistent with OFFα RGC physiology in rd10 retina (Margolis et al., 2008; Werginz et al., 2025). Thus, we conclude that the cells in our original dataset with an index below −0.5 represent the OFFα RGC subtype and are unique in their responses to electrical stimulation pulses. We refer to OFF-responding OFF RGCs as OFFα RGCs for the remainder of the manuscript.

It is widely accepted that there are four types of α RGCs, ONt (transient), ONs (sustained), OFFt and OFFs in mouse (Krieger et al., 2017). Within the OFFα dataset, we identified cells with distinct responses to electrical stimulation, which we believed to be sustained and transient (Fig. 2*F*). Recent work has shown OFFt and OFFs α RGCs can be differentiated based on their physiological response properties (Werginz et al., 2025). In order to determine if these cells were transient and sustained cells, we performed a *k*-means clustering algorithm with three physiological input variables (action potential width, peak firing rate, and time to peak firing rate). Based on these parameters, two distinct clusters were identified. We found the cells within the cluster we believed to be transient OFFα RGCs had briefer action potentials than the cluster we believed to be sustained, consistent with past work (Werginz et al., 2025; Fig. 2*G*,*H*, *t* test: *t*_(12.37)_ = 4.94, *p* < 0.0005). Furthermore, the transient cluster had a significantly higher peak firing rate compared with the sustained cluster (Murphy and Rieke, 2011; Werginz et al., 2025; Fig. 2*I*, *t* test: *t*_(8.07)_ = −2.75, *p* < 0.05). Interestingly, transient and sustained cells also displayed different response kinetics to the stimulation pulse train. Sustained cells had a significantly longer time to peak response (Fig. 2*J*, *t* test: *t*_(9.52)_ = 3.56, *p* = 0.005). Taken together these results demonstrate correct OFF electrical responses occur in both subtypes of OFFα RGC while maintaining physiology that broadly aligns with previous descriptions of transient and sustained OFFα RGCs.

### Frequency and intensity tuning of ON and OFF responses

**Figure 3.**
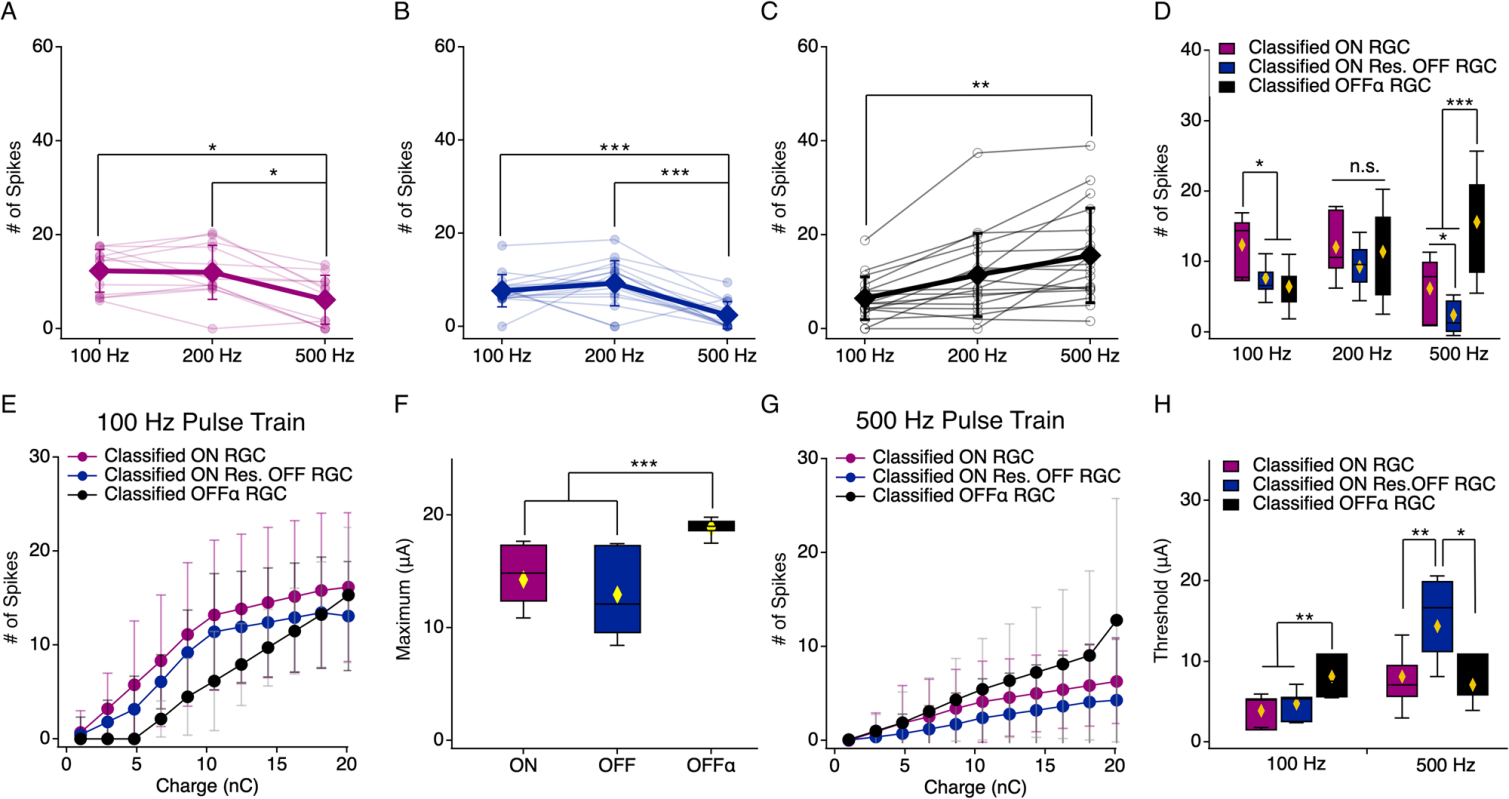
Pulse train frequency and intensity encoding in ON and OFF RGCs. ***A***, Average number of evoked spikes for a 100, 200, and 500 Hz pulse train for ON RGCs, *n* = 13. ***B***, Same as ***A*** but for ON-Responding OFF RGCs, *n* = 23. ***C***, Same as ***A*** but for OFFα RGCs *n* = 18. ***D***, Comparison of average # of spikes between ON (magenta), ON-responding OFF (blue), and OFFα cells to 100, 200, and 500 Hz stimuli. ***E***, Average intensity response curves for ON-responding ON, OFF, and OFFα cells to a 100 Hz pulse train. ***F***, Box plot comparison of the current value that maximum spiking is reached for ON, OFF, and OFFα cells to a 100 Hz pulse train. ***G***, Same as ***E*** but for a 500 Hz pulse train. ***H***, Comparison of intensity response threshold for ON, OFF, and OFFα cells for 100 and 500 Hz pulse trains. Significance levels were as follows: *p < 0.05, **p < 0.005, and ***p < 0.0005. Box plots use the Tukey 1st quartile, median, and 3rd quartile, whiskers represent 1 SD, and yellow diamonds represent the mean. For the entire figure, *n* = 19 animals (P81–161), mean age = 119 ± 28.

Past work has demonstrated that spiking responses for unidentified RGCs are modulated by both pulse train frequency and the charge magnitude of the pulse, although response functions tend to be variable between individual RGCs (Lee and Im, 2019; Damle et al., 2021; Lee et al., 2024). This indicates that specific stimulation paradigms may be capable of selectively driving some information pathways. To determine if ON RGCs, ON-responding OFF RGCs, and OFFα RGCs are modulated differently by these stimulation parameters, we stimulated these three RGC groups with a single pulse and 100, 200, and 500 Hz pulse trains. We also varied pulse charge from 1 nC to 20 nC per pulse (Fig. 3*E–H*).

In Figure 1 we showed that ON RGCs and ON-responding OFF RGCs had a prominent depolarization and action potential firing during and immediately following the stimulation pulse(s), hallmarks of an electrically evoked ON response (Fig. 1*I*,*J*). In contrast, OFFα RGCs were hyperpolarized during stimulation and subsequently fired action potentials with a delay of 180–280 ms after the end of the stimulus, consistent with an OFF response (Fig. 2*D*). Thus, we measured responses within the relevant physiological response window for each of the groups. We found a nonlinear response relationship for pulse train frequency for ON responses with significantly less action potentials in response to 500 Hz stimulation compared with 100 and 200 Hz stimulation for both ON RGCs (Fig. 3*A*, one-way ANOVA: *F*_(2,37)_ = 5.49, *p* < 0.05), post hoc Tukey HSD comparisons: ps’ < 0.05 and ON-responding OFF RGCs (Fig. 3*B*, one-way ANOVA: *F*_(2,49)_ = 14.47, *p* < 0.0005, post hoc Tukey HSD comparisons: ps’ < 0.05). In contrast, OFF responses originating in OFFα RGCs increased linearly as pulse train frequency increased, with significantly more spikes occurring at 500 Hz compared with 100 Hz pulse trains (Fig. 3*C*, one-way ANOVA: *F*_(2,53)_ = 5.63, *p* < 0.05, post hoc Tukey HSD comparisons: ps’ < 0.05). In both the 100 and 500 Hz conditions, ON RGCs had significantly more spikes than ON-responding OFF RGCs (Fig. 3*D*, one-way ANOVA: *F*_(2,47)_ = 7.87, *p* < 0.005, post hoc Tukey HSD comparisons: ps’ < 0.05). Additionally, OFFα RGCs displayed significantly more spikes than the ON-responding groups to a 500 Hz pulse train (Fig. 3*D*, one-way ANOVA: *F*_(2,45)_ = 15.73, *p* < 0.0005, post hoc Tukey HSD comparisons: ps’ < 0.05). We did not find any differences between sustained and transient OFFα RGCs (Fig. S1); thus we pooled data from both sustained and transient OFFα RGCs in all subsequent analyses; however separation for all subsequent analysis can also be found in Figure S1. These results demonstrate that response characteristics systematically differ between ON- and OFF-responding RGCs even though the ON-responding group contains both morphological ON and OFF RGCs.

When examining charge response relationships, we found that all groups generally increased spiking as charge increased across both 100 and 500 Hz frequencies (Fig. 3*E*,*G*). However, for a 100 Hz pulse train, the intensity response function was similar for both ON RGCs and ON-responding OFF RGCs, saturating before reaching maximal charge delivery, similar to prior results for single pulses (Damle et al., 2021; Fig. 3*E*). For OFF-responding OFFα RGCs, responses increased linearly with their spiking maximum occurring at significantly higher current values than the other two groups (Fig. 3*F*, one-way ANOVA: *F*_(2,40)_ = 9.13, *p* = 0.0005, post hoc Tukey HSD comparisons: ps’ < 0.05). However, charge threshold was significantly higher for OFFα RGCs than for ON-responding group (Fig. 3*H*, one-way ANOVA: *F*_(2,42)_ = 11.28, *p* = 0.0001, post hoc Tukey HSD comparisons: ps’ < 0.05). For 500 Hz stimulation, responses increased linearly across all groups, although OFF responses increased more with charge leading to greater spiking for OFF responses at 500 Hz than ON responses in ON RGCs and ON-responding OFF RGCs. Intriguingly, ON-responding OFF RGCs showed a significantly increased charge threshold for spiking to 500 Hz pulse trains, compared with ON RGCs and OFFα RGCs (Fig. 3*H*, one-way ANOVA: *F*_(2,55)_ = 10.03, *p* = 0.0002, post hoc Tukey HSD comparisons: ps’ < 0.05). Taken together, these results suggest that 500 Hz stimulation may maximize differences between correct OFF responses and inappropriate ON responses in OFF RGCs. Therefore, we will focus on 500 Hz stimulation for the remainder of the manuscript.

### Presynaptic circuitry gives rise to electrically evoked OFF responses

**Figure 4.**
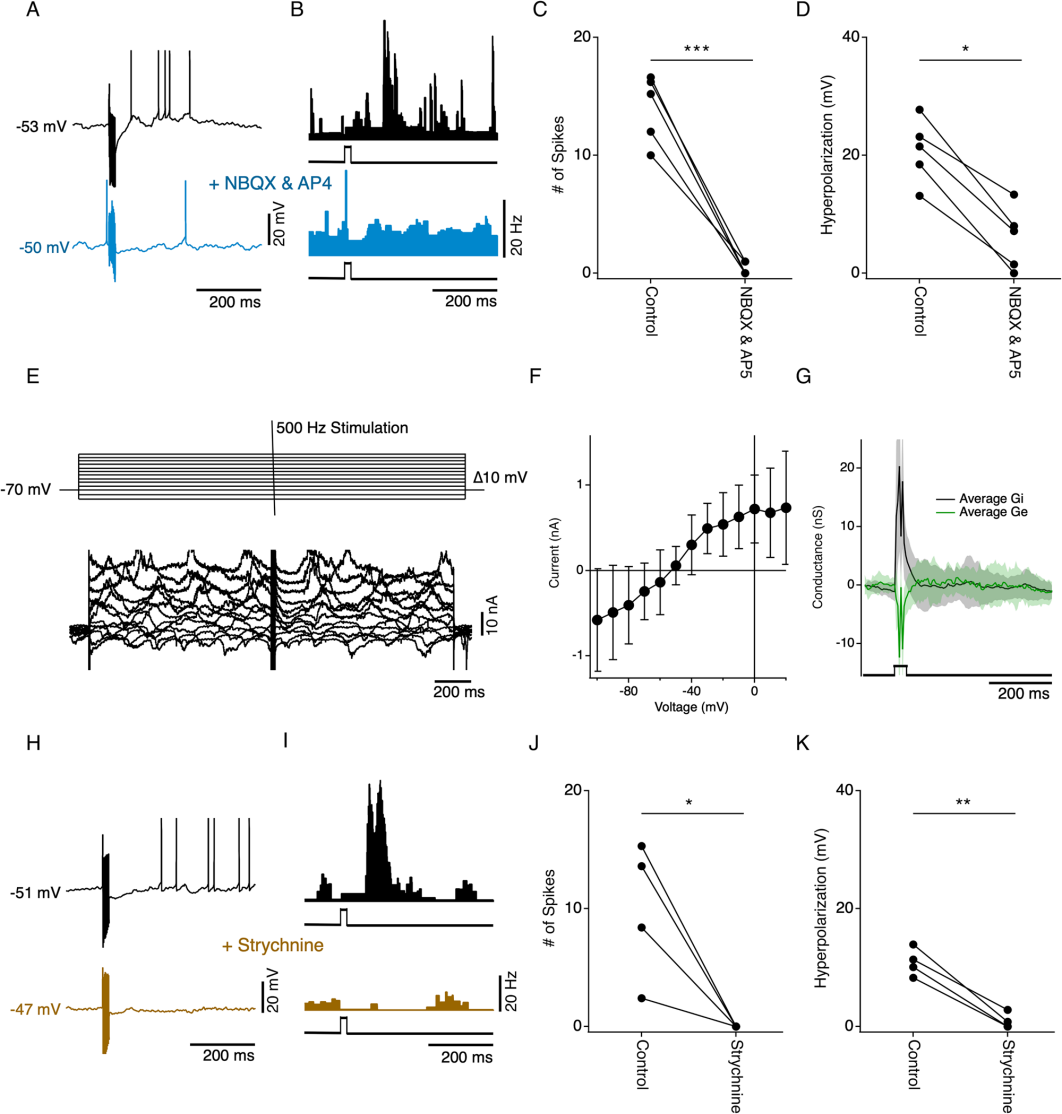
OFF responses in OFFα RGCs are not due to presynaptic glutamate. ***A***, Example current-clamp trace of OFFα RGC in control (black) and with the addition of glutamate antagonists NBQX and AP4 (blue). ***B***, Average PSTH in response to a 500 Hz stimulus for control and NBQX and AP4 (*n* = 5, # of animals 4, P73–114). ***C***, Change in number of spikes from control to NBQX and AP4. ***D***, Change in peak hyperpolarization from control to NBQX and AP4. ***E***, Voltage-step paradigm. Voltage from −100 to +20 mV. A 500 Hz stimulation pulse train occurred halfway through the voltage step (top). Example currents recorded at each voltage step (bottom). ***F***, Average *I*–*V* curve for recorded cells (*n* = 10, # of animals 5, P93–152). Error bars represent 1 SD. ***G***, Average excitatory and inhibitory conductance. Shading represents 1 SD. ***H–K***, Same as ***A–D*** but for the addition of the glycine antagonist strychnine (orange; *n* = 4, # of animals 3, P65–95). Significance levels were as follows: *p < 0.05, **p < 0.005, ***p < 0.0005 and +*p* < 0.1, paired *t* test.

In healthy retina, OFF responses are formed by OFF bipolar cells, which are depolarized when light decreases. In retinal prosthetics increases in light are translated into electrical stimulation, which would correspond to an ON response. The question of how a decrease in electrical stimulation could generate an appropriate and selective OFF response in the correct OFF RGCs in photoreceptor degenerated retina remains unsolved. While subretinal stimulation should primarily affect the inner nuclear layer, prior studies have shown direct activation of RGCs can also occur (Tsai et al., 2009; Jensen, 2012; Boinagrov et al., 2014). Therefore, these electrically evoked OFF responses could arise in OFFα RGCs because of (1) unique presynaptic network activation or because (2) the electrical stimulation uniquely interacts with the intrinsic properties of the OFFα RGC. To determine if OFFα RGCs require network activation for OFF responses, we blocked presynaptic excitatory inputs using glutamate receptor antagonists AP5 and NBQX. We found that glutamate receptor block eliminated OFFα RGC stimulus-evoked spikes, demonstrating that presynaptic inputs are required to generate electrically evoked OFF responses (Fig. 4*A–C*, paired *t* test: *t*_(4)_ = 9.88, *p* = 0.0005). However, hyperpolarization evoked by stimulation was also reduced under glutamatergic blockade (Fig. 4*A*,*D*, paired *t* test: *t*_(4)_ = 4.97, *p* < 0.05). This indicates that the inhibitory input, likely originating from an amacrine cell, is driven via a presynaptic glutamate source, and the loss of this inhibition may have an impact on OFF responses in OFFα RGCs.

Since blocking glutamatergic excitation also led to a loss of hyperpolarization in OFFαs, there were two possible interpretations to our results: (1) reduced excitatory drive from OFF bipolar cells following the stimulation pulse train resulted in the loss of OFF spiking or (2) inhibition is required to drive spiking through a rebound excitation mechanism (Margolis et al., 2008; Biel et al., 2009). To dissect these two possibilities, we performed a subset of experiments in voltage clamp to measure the stimulation-evoked excitatory and inhibitory synaptic conductances to OFFα RGCs (Fig. 4*E–G*). We found stimulation-evoked currents reversed at the chloride reversal potential indicating that all presynaptic inputs onto OFFα RGCs are inhibitory (Fig. 4*F*). At the time of the OFF spiking response observed in voltage recordings, inhibitory conductance had decayed back to baseline (Fig. 4*G*), leaving no inhibitory or excitatory conductance that could account for depolarization and spiking. In contrast, ON-responding OFF RGCs receive significantly more excitation during the stimulus pulse train (Fig. S2*A,B,D*, *t* test: *t*_(7)_ = 3.36, *p* < 0.05). These data in combination with the glutamatergic blockade suggest OFF bipolar cell mediated synaptic excitation is not driving electrically evoked OFF responses in OFFα RGCs. Likely, a presynaptic inhibitory input which is driven by glutamate is responsible for generating some form of postsynaptic excitation that is absent in the voltage-clamp experiments, presumably a voltage-gated conductance in the OFFα RGCs.

To target the input from this inhibitory driver, we applied the glycinergic antagonist strychnine. Although both GABAergic and glycinergic inhibition are present in the retina and OFFα RGCs express both GABA and glycine receptors, past work has shown that OFFα RGCs receive direct somatic glycinergic inhibition from AII amacrine cells that contribute significantly to light evoked signaling in these cells(Grimes et al., 2022). The addition of strychnine eliminated pulse train-evoked hyperpolarization (Fig. 4*H*, paired *t* test: *t*_(3)_ = 8.99, *p* < 0.005) as well as the OFF spiking response following the pulse train (Fig. 4*E–G*, paired *t* test: *t*_(3)_ = 3.415, *p* = 0.041). These results confirm that the electrically evoked OFF response is caused by rebound excitation following inhibition.

**Figure 5.**
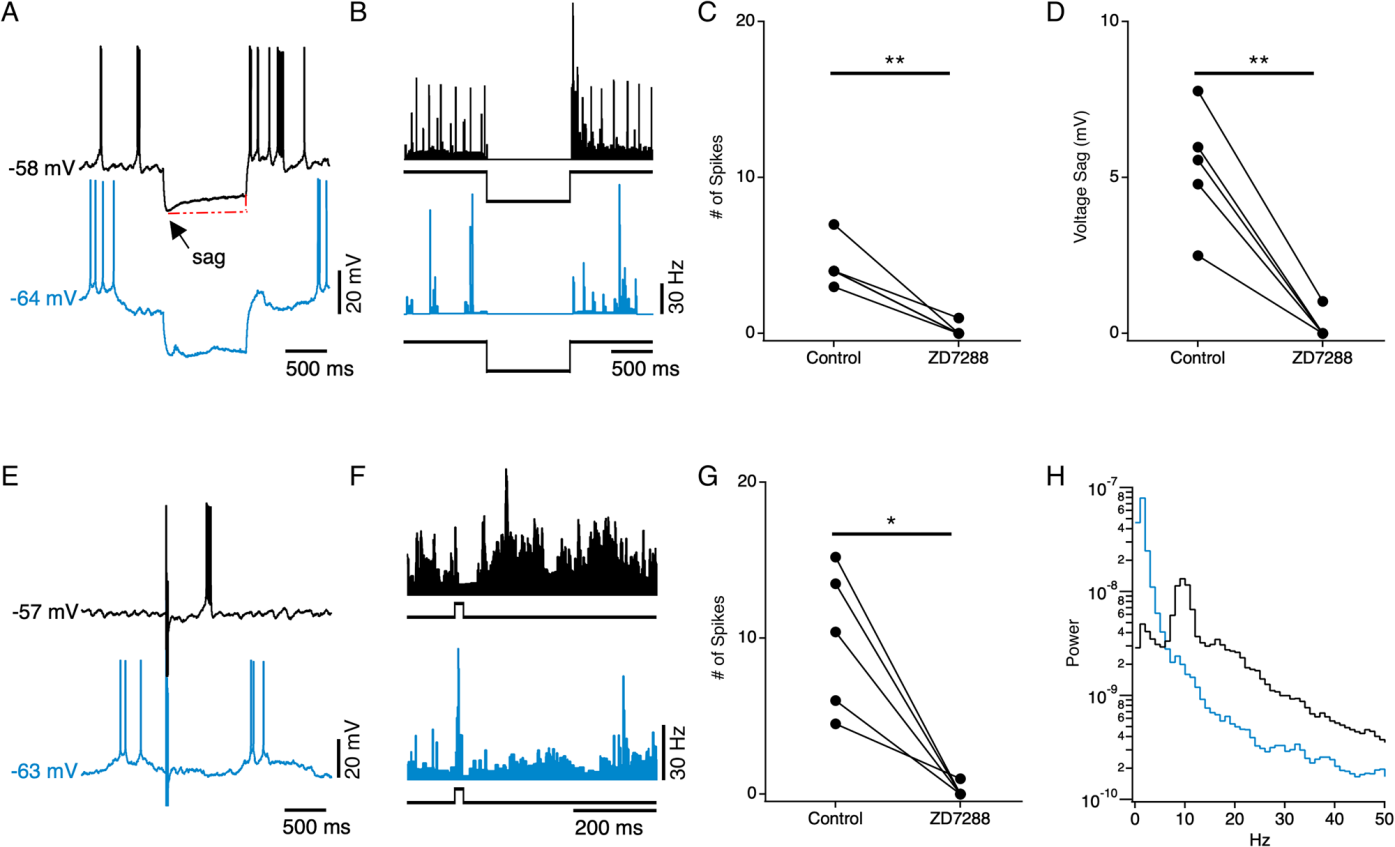
HCN channels mediate rebound firing in OFFα RGCs. ***A***, Example traces of a 125 µA hyperpolarizing current step. ***B***, Average PSTH for the current step in ***A*** (*n* = 5). ***C***, Change in number of spikes from control to ZD7288 for hyperpolarizing current injection. ***D***, Change in voltage sag from control to ZD7288. ***E***, Example trace in response to a 500 Hz pulse train. ***F***, Average PSTH for the 500 Hz pulse train (*n* = 5). Control data is in black, ZD7288 data is in blue. ***G***, Same as ***C*** but for a 500 Hz pulse train. ***H***, Average PSD for control (black) and ZD7288 (blue). Significance levels were as follows: *p < 0.05, **p < 0.005, paired *t* test (***C***, ***D***, ***G***). For the entire figure, *n* = 4 animals, P78–207.

PIRE is a well-documented mechanism that would allow for a delayed increase in spiking driven by inhibition, which has been shown to be mediated by hyperpolarization-activated cyclic nucleotide (HCN) channels (Biel et al., 2009). To test if HCN channels were necessary for the electrically evoked OFF responses, we again recorded from OFFα RGCs in current clamp and measured rebound spiking in response to hyperpolarizing current steps and 500 Hz pulse trains. OFFα RGCs displayed both physiological signatures of HCN channel activation—the voltage sag and rebound spiking—in response to a hyperpolarizing step (Fig. 5*A*). To determine if HCN channels were responsible, we applied 20 µM ZD7288, a selective HCN channel antagonist (BoSmith et al., 1993). Rebound excitation was reduced or eliminated with the application of ZD7288 (Fig. 5*A–C*, paired *t* test: *t*_(4)_ = 5.93, *p* < 0.005) and *I*_h_ sag current was also significantly reduced (Fig. 5*D*, paired *t* test: *t*_(4)_ = 5.78 *p* < 0.005). Resting membrane potential in ZD7288 was also increased (60.76 ± 6 mV to 67.84 ± 5.8 mV, *n* = 5), indicating an HCN channel contribution to resting membrane potential. These results are consistent with other reports of HCN function in OFFα RGC physiology (Margolis et al., 2008). Importantly, application of ZD7288 also eliminated electrically evoked OFF responses to 500 Hz stimulation pulse trains (Fig. 5*E–G*, paired *t* test: *t*_(4)_ = 4.67, *p* < 0.05). We also found that spontaneous activity in OFFα RGCs shifted from 10 to 1–2 Hz in ZD7288 (Fig. 5*H*), consistent with past work (Trenholm et al., 2012). Taken together, these results show that electrically evoked OFF responses in OFFα RGCs are driven by a classic HCN channel mediated PIRE mechanism following stimulation-evoked hyperpolarization, rather than a direct excitatory mechanism.

### AII amacrine cells are required for OFFα RGC responses

**Figure 6.**
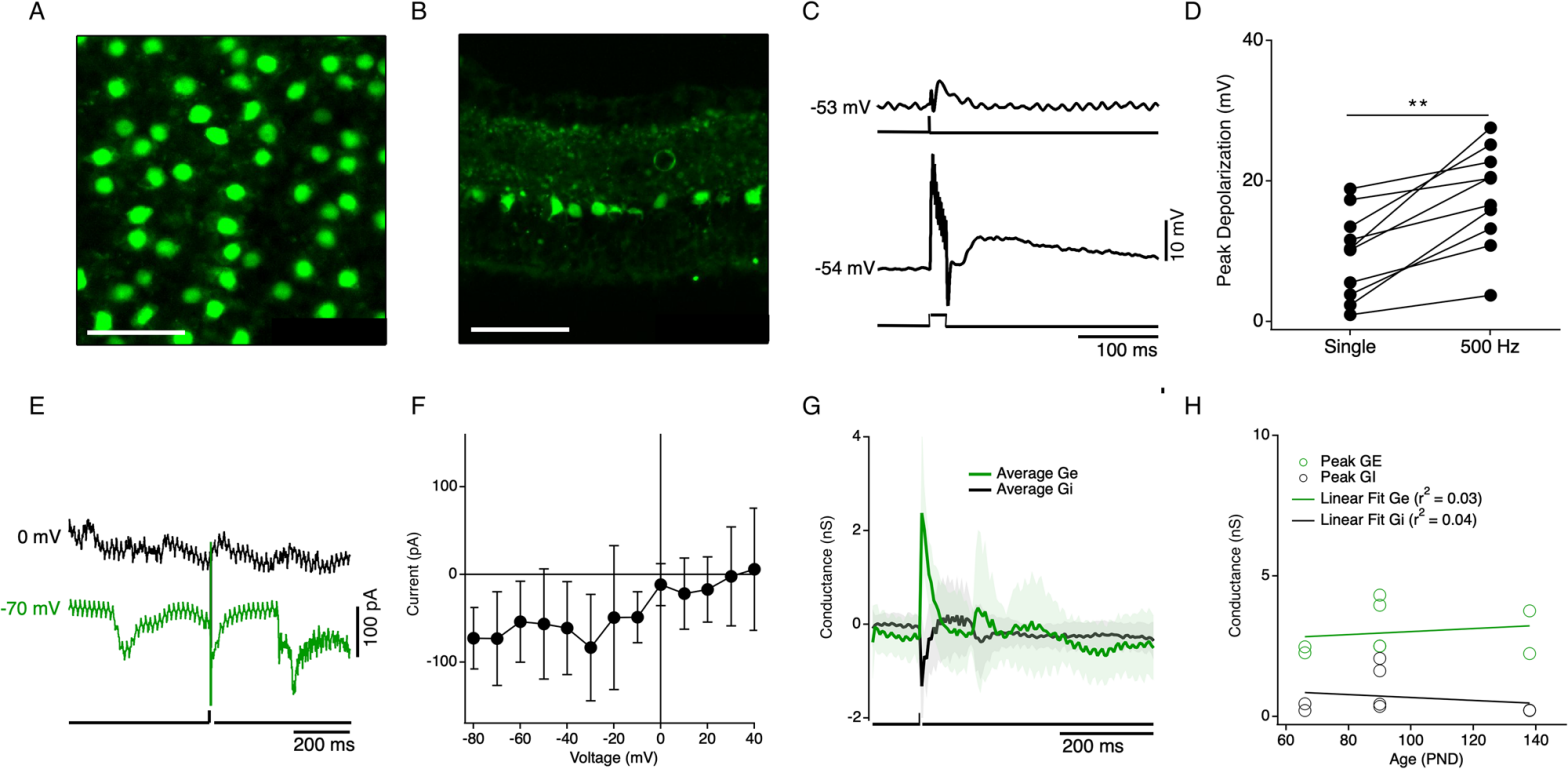
AII amacrine cells are activated via electrical stimulation. ***A***, GFP-positive AII amacrine cells in degenerated retina imaged en face and (***B***) vertical slice. ***C***, Example voltage traces from an AII amacrine cell in response to a single pulse and 500 Hz pulse train. ***D***, Peak stimulus-evoked depolarization for single pulse and 500 Hz pulse train (*n* = 10, # of animals 4, P66–143). ***E***, Excitatory (black) and inhibitory (green) currents in response to a single electrical pulse. ***F***, Average *I*–*V* curve for recorded cells (*n* = 8). Error bars represent 1 SD. ***G***, Average excitatory and inhibitory conductance. Shading represents 1 SD. ***H***, Peak excitatory and inhibitory conductance plotted against age. Dots represent each cell. Lines represent the linear fit. Scale bar, 50 µm. Significance levels were as follows: **p < 0.005, paired *t* test (***D***). For ***E–H*** # of animals 3, P66–140.

Since inhibition rather than excitation mediated OFF responses in photoreceptor degenerated retina, we were interested in identifying the source(s) of presynaptic inhibition that underlie electrically evoked OFF responses, which has important implications for the design of retinal prosthetics. Connectomics studies have shown OFFα RGCs receive significant input from AII amacrine cells (Marc et al., 2014). To determine if AIIs are involved in generating electrically evoked OFF responses in OFFα RGCs, we made whole-cell recordings from AIIs in whole-mount rd10 retina. To target AIIs in the degenerated retina, we crossed the CDH1-eGFP (Gong et al., 2003; Siegert et al., 2009) mouse, which selectively express GFP in AII amacrine cells (Firl et al., 2015), with the rd10 mouse to introduce the *Pde6b^rd10^* allele (Chang et al., 2002; Fig. 6*A*). Although prior anatomical studies showed AII disorganization starting at PND 40 (Barhoum et al., 2008), we found cells persisted and did not undergo secondary cell death after degeneration was complete (Fig. 6*A*,*B*, PND 105). All AII intracellular recordings were performed after PND 60 (PND 66-143). When stimulated with either a single pulse or 500 Hz pulse train, AII amacrine cells displayed a prominent depolarization during and immediately following electrical stimulation (Fig. 6*C*). For all cells, 500 Hz stimulation depolarization was greater than single pulse stimulation (Fig. 6*D*, paired *t* test: *t*_(9)_ = 5.23, *p* = 0.005). This work represents the first demonstration of electrical activation of AII amacrine cells.

In healthy retina, AII amacrine cells are involved in signal formation at scotopic (rod pathway) and photopic (ON pathway) light levels. A long prevailing theory is that the degenerated retina is in a “scotopic state” due to increased gap junctional coupling of the AII network (Ivanova et al., 2015). However, no prior work has looked at activation of the Rod BC-AII pathway to electrical stimulation. Since we found the presynaptic inhibitory driver of OFFα responses was in turn driven by a glutamatergic source, we hypothesized that AII amacrine cells were receiving excitation via electrical activation of rod bipolar cells (RBCs). To test this, we recorded from AII’s in voltage clamp and stimulated with a single electrical pulse. We found electrically evoked currents were primarily excitatory reversing around the cation reversal potential (Fig. 6*E*,*F*). A small amount of inhibitory conductance was observed with a slower time to peak (Fig. 6*G*). Importantly, we found excitatory and inhibitory inputs to AII amacrine cells were not altered as degeneration progressed (Fig. 6*H*). Thus, glutamatergic input from the rod pathway drives AII depolarization which in turn may elicit OFF responses in OFFα RGCs.

**Figure 7.**
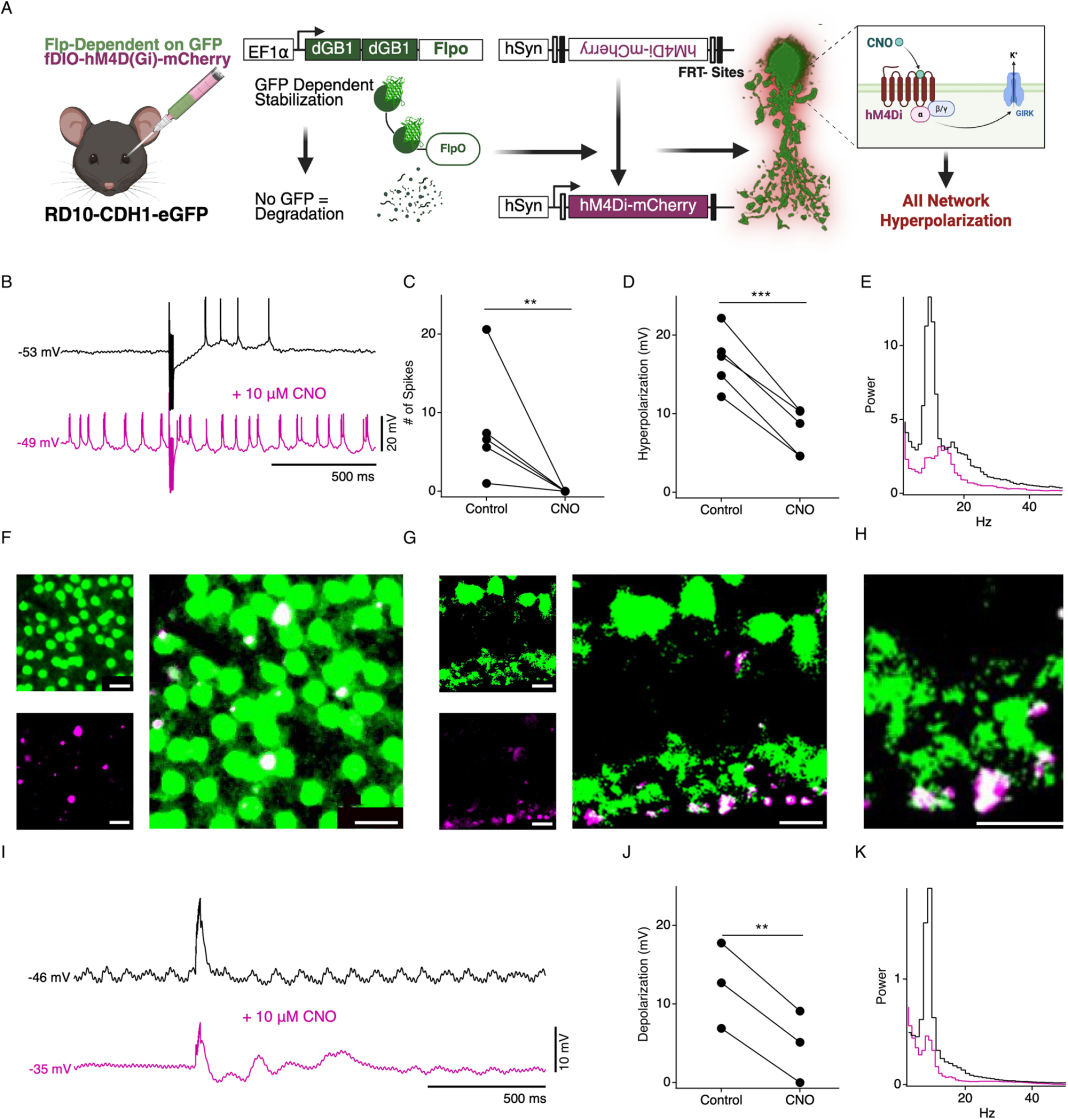
AII amacrine network inhibition is necessary for PIRE. ***A***, Schematic combinatorial strategy using the FLP-DOG/DREADD(Gi) system. Dual injection of FLP-DOG and hM4D(Gi)-mCherry were injected into RD10-CDH1-eGFP mice. ***B***, Example current-clamp trace of OFFα RGC in control (black) and with the addition of CNO (Pink). ***C***, Change in number of spikes from control to CNO. ***D***, Change in peak hyperpolarization from control to CNO (# of animals = 3, P85–128). ***E***, Average PSD for OFFα RGCs in control (*n* = 9) and CNO (*n* = 5). ***F***, En face images of mCherry (magenta) and GFP (green) colocalization. ***G***, Vertical slice image of mCherry (magenta) and GFP (green) colocalization. ***H***, Zoomed in view of dendrites from ***G***. ***I***, Example current-clamp trace of AII amacrine cell in control (black) and with the addition of CNO (pink). ***J***, Change in peak depolarization in AII amacrine cells from control to CNO (*n* = 3, # of animals = 3, P92–128). ***K***, Average PSD for AII amacrine cells in control (*n* = 11) and CNO (*n* = 3). Scale bar, 20 µm. Significance levels were as follows: **p < 0.005, and ***p < 0.0005, paired *t* test (***C***, ***D***, ***J***).

The role of any specific amacrine cell in shaping electrical responses in photoreceptor degenerated retina is completely unknown. To determine if AII amacrine cell activation was required to elicit electrically evoked OFF responses in OFFα RGCs, we suppressed AII amacrine cells using a GFP-dependent combinatorial genetic approach to selectively express inhibitory DREADDS in the GFP expressing AII amacrine cells. We simultaneously intravitreally injected AAV1 FLP-recombinase dependent on GFP (FLP-DOG; Tang et al., 2016, 2017) in the CDH1-eGFP-RD10 mice to drive expression of FLP-recombinase selectively in GFP expressing AII amacrine cells and AAV8 flpase-dependent inhibitory DREADD fDIO-hM4Di-mCherry. This resulted in inhibitory DREADD expression in a subset of dually transfected AII's (Fig. 7*A*). Ten to 12 d post injection, we made whole-cell recordings from OFFα RGCs. We first recorded responses to 500 Hz pulse train stimulation in control conditions, followed by application of 10 µM CNO in the superfusate. Fifteen minutes after beginning CNO application, we again recorded responses to 500 Hz stimulation. We found rebound firing was eliminated following addition of CNO in OFFα RGCs (Fig. 7*B*), consistent with an AII-mediated PIRE mechanism for the electrically evoked OFF response in OFFα RGCs. When spontaneous spikes were subtracted from the stimulation-evoked window, there was a complete reduction in all cells examined (Fig. 7*C*, paired *t* test: *t*_(4)_ = 8.03, *p* < 0.005). OFFα hyperpolarization during the pulse train was significantly reduced but not eliminated, similar to the results we obtained in glycinergic blockade (Fig. 7*D*, paired *t* test: *t*_(4)_ = 10.45, *p* < 0.0005). In addition to the elimination of the electrically evoked OFF response, the resting membrane potential also increased from −53.84 ± 1.98 mV to −45.52 ± 4.76 mV and basal firing increased. Despite more spontaneous firing, we found that spontaneous spikes were less structured temporally, with a 78% reduction of peak oscillatory power and a shift in peak frequency from 10 to 15 Hz (Fig. 7*E*). This is consistent with a role for the AII amacrine network acting as a pacemaker (Biswas et al., 2014) and providing both phasic and tonic inhibition to the OFFα (Münch et al., 2009; Kim et al., 2020). Taken together these results demonstrate the key role the AII amacrine cell plays in generating electrically evoked OFF responses.

To confirm that the action of CNO was mediated by AIIs, we first imaged mCherry and GFP expression in these retinas to confirm that DREADD fDIO-hM4Di-mCherry was expressed in AIIs. We observed mCherry and GFP colocalization primarily in AII dendritic arbors and only observed sparse mCherry expression in cell bodies (Fig. 7*F*). Given that our approach requires two overlapping viral transfections, it is likely only a subset of AIIs will be transfected to express DREADD. Unfortunately, given the lack of mCherry expression in cell bodies, it is not possible to quantify the percent of AIIs expressing DREADD. Regardless, we did observe colocalization in the dendrites of AIIs (Fig. 7*G*,*H*). Given that AIIs form a gap junction coupled network, a subset of AII’s may be sufficient to reduce AII network output. To confirm this, we recorded from AII amacrine cells blind to their DREADD expression status to determine the effect of DREADD mediated inhibition on the network. AII resting membrane potential hyperpolarized in CNO treatment (−45.3 ± 3.5 mV to −50.2 ± 11 mV, *n* = 3). Importantly, electrically evoked depolarization was reduced, although not eliminated (Fig. 7*I*,*J*, paired *t* test: *t*_(2)_ = −14.98, *p* < 0.005). Taken together these results indicate that CNO is acting to reduce excitability of AIIs, as expected, even if complete suppression of stimulation-evoked activity was not achieved. This provides further confirmation of the AII amacrine as a key presynaptic partner to OFFα RGCs.

## Discussion

The goal of vision restoration is to generate physiologically relevant activity in the visual pathways, such as appropriately timed ON and OFF signals in their respective pathways; however, the extent to which ON and OFF RGCs maintain their response polarity for electrical stimulation remains largely unclear. Past work has relied heavily on extracellular recording techniques, which can only sample from RGCs that reach spike threshold; therefore, weakly or nonresponding RGCs will be missed. Here we show this is a relevant limitation, as we found about half of all RGCs sampled did not respond to a single 20 nC biphasic pulse and that all nonresponding RGCs had OFF morphology. While brief stimulation does not stimulate OFF RGCs, high-frequency stimulation in the 100–500 Hz range can drive responses in these cells. However, the majority of OFF RGCs respond during and immediately following the stimulation pulse train, indicative of an ON-polarity response. Although it has been widely assumed that electrical stimulation should indiscriminately stimulate both ON and OFF bipolar cells and generate ON responses in both ON and OFF RGCs (Goetz et al., 2015; Werginz et al., 2015; Goetz and Palanker, 2016; Golden et al., 2019; Paknahad et al., 2022), this is the first time this has been explicitly tested.

Intriguingly, a small subset of OFF RGCs, maintained a functional OFF response following termination of the stimulation pulse train. We found these OFF-responding OFF cells were OFFα RGCs and that rebound excitation following AII-mediated inhibition was required to generate these appropriate OFF responses. OFFα RGCs can be further divided into sustained and transient subtypes. We found our dataset contained an equal proportion of sustained [47.4% (18/38)] and transient [52.6% (20/38)] OFFα RGCs. Interestingly, while prior reports have shown differences in the response thresholds of transient and sustained cells in response to light (Pang et al., 2003; Krieger et al., 2017), here we find no significant difference between the subtypes in response to electrical pulse trains (Fig. S1*E–H*). This may be due to the different mechanisms engaged in response to light and electrical stimulation. In the healthy retina, differences in firing properties of sustained and transient RGCs have been attributed to different presynaptic inputs (Awatramani and Slaughter, 2000) and intrinsic properties (Werginz et al., 2025). Additionally, the transient subtype shows further heterogeneity along the dorsal-ventral axis, with responses increasing in duration in the dorsal retina (Warwick et al., 2018; Werginz et al., 2020). These changes in response characteristics have been primarily attributed to differential inputs from the rod pathway via the AII amacrine cell (Pang et al., 2003; Warwick et al., 2018). However these findings have not been attributed to direct outputs to OFFα which are consistent between sustained and transient RGCs (Grimes et al., 2022). Here, using pharmacological and DREADD manipulations, we found both cell types require the same pathway and mechanism to produce electrical OFF responses (Fig. S1*I–K*). It is possible that some of the heterogeneity observed in OFFα responses is due to dorsal-ventral bias, but heterogeneity in electrically spiking is not significantly different between the two cell types [Bartlett's test, *χ*^2^(1, *N* = 37) = 0.27, *p* > 0.05]. It seems likely that AII amacrine cells may be uniquely positioned to generate electrically evoked OFF responses in OFFα RGCs as they have been reported to form selective synapses onto the laminating dendrites in the OFF layer and somas in the ganglion cell layer of both OFFt-α and OFFs-α RGCs, but not with other OFF RGC subtypes (Manookin et al., 2008; Murphy and Rieke, 2008; Wyk et al., 2009; Buldyrev et al., 2012; Grimes et al., 2022).

In addition to demonstrating electrically evoked OFF responses in a defined OFF RGC subtype, we identified the circuit elements responsible for this unique electrical OFF response. Strong inhibitory input from AIIs is critical for generating OFF responses, as electrically evoked OFF responses were completely blocked when AIIs were chemogenetically inhibited. Our earlier work showed that OFF RGCs in photoreceptor degenerated retina receive stimulation-evoked inhibition from multiple inhibitory sources (Carleton and Oesch, 2024). In the present study, we show that AII amacrine cells are activated by electrical stimulation; however, our understanding of how the AII is activated by electrical stimulation remains incomplete. Three potential pathways may contribute: glutamatergic input from RBCs, gap junction input from ON-cone bipolar cells, and direct electrical activation of the AII itself. Our finding that hyperpolarization and PIRE are reduced during glutamatergic blockade suggests a major role for RBCs in driving AII-mediated inhibition of OFFα RGCs. However, our recent data also indicate that gap junction input can reduce inhibitory currents to OFF RGCs generally, suggesting cone bipolar cells could play a secondary role(Carleton and Oesch, 2024). Furthermore, while inhibition of the AII network with DREADDs and application of glycine resulted in the loss of OFF spiking and a significant reduction in electrically induced hyperpolarization, there was still a remaining GABAergic inhibitory component, albeit small, that likely contributes to observed OFF responses. Regardless, this work demonstrates that some amacrine cells are activated by electrical stimulation and shows that complex inhibitory signaling can be evoked by electrical stimulation, as opposed to simple feedforward excitation. Furthermore, it identifies a specific role for AIIs generating RGC responses to electrical stimulation.

Although OFF responses are only found in OFFα RGCs, which are a small subset of the total OFF RGCs in the mouse retina, recent work suggests that these may be orthologs of the dominant RGC types that comprise the majority of RGCs in human fovea, the midget, and parasol cells (Hahn et al., 2023). These observations combined with work showing that the AII network in primate fovea is largely consistent with work done in mouse (Strettoi et al., 2018) provides strong support for the idea that human retina will respond to electrical stimulation similarly. Patients with prosthetic devices do not report dark phosphenes or perception of negative contrast and perform significantly worse with negative contrast stimuli (Greenwald et al., 2009; Palanker et al., 2020; Holz et al., 2025). Given the prominent role of the OFF pathway in forming high acuity spatial vision (Tyler et al., 1992; Mazade et al., 2019), stimulation of only the ON pathway significantly restricts the quality of restored vision and generating appropriate OFF responses in retinal prosthetic stimulation will clearly be advantageous (Golden et al., 2019). Although more work remains to show how the stimulation patterns reported here operate in human retina and across disease states, this work lays a physiological framework to understand how OFF responses can be restored to the OFF pathway in blind patients.
